# Prediction and Variable Selection in High-Dimensional Misspecified Binary Classification

**DOI:** 10.3390/e22050543

**Published:** 2020-05-13

**Authors:** Konrad Furmańczyk, Wojciech Rejchel

**Affiliations:** 1 Institute of Information Technology, Warsaw University of Life Sciences (SGGW), Nowoursynowska 159, 02-776 Warszawa, Poland; 2 Faculty of Mathematics and Computer Science, Nicolaus Copernicus University, Chopina 12/18, 87-100 Toruń, Poland; wrejchel@gmail.com

**Keywords:** misclassification risk, model misspecification, penalized estimation, supervised classification, variable selection consistency

## Abstract

In this paper, we consider prediction and variable selection in the misspecified binary classification models under the high-dimensional scenario. We focus on two approaches to classification, which are computationally efficient, but lead to model misspecification. The first one is to apply penalized logistic regression to the classification data, which possibly do not follow the logistic model. The second method is even more radical: we just treat class labels of objects as they were numbers and apply penalized linear regression. In this paper, we investigate thoroughly these two approaches and provide conditions, which guarantee that they are successful in prediction and variable selection. Our results hold even if the number of predictors is much larger than the sample size. The paper is completed by the experimental results.

## 1. Introduction

Large-scale data sets, where the number of predictors significantly exceeds the number of observations, become common in many practical problems from, among others, biology or genetics. Currently, the analysis of such data sets is a fundamental challenge in statistics and machine learning. High-dimensional prediction and variable selection are arguably the most popular and intensively studied topics in this field. There are many methods trying to solve these problems such as those based on penalized estimation [1,2]. The main representative of them is Lasso [3], that relates to $l_{1}$-norm penalization. Its properties in model selection, estimation and prediction are deeply investigated, among others, in [2,4,5,6,7,8,9,10]. The results obtained in the above papers can be applied only if some specific assumptions are satisfied. For instance, these conditions concern the relation between the response variable and predictors. However, it is quite common that a complex data set does not satisfy these model assumptions or they are difficult to verify, which leads to the fact that the considered model is specified incorrectly. The model misspecification problem is the core of the current paper. We investigate this topic in the context of high-dimensional binary classification (binary regression).

In the classification problem we are to predict or to guess the class label of the object on the basis of its observed predictors. The object is described by the random vector (X,Y), where $X\in R^{p}$ is a vector of predictors and Y∈{−1,1} is the class label of the object. A classifier is defined as a measurable function $f:R^{p}\to R,$ which determines the label of an object in the following way:

iff(x)≥0,thenwepredictthaty=1.

Otherwise, we guess that y=−1.

The most natural approach is to look for a classifier f, which minimizes the misclassification risk (probability of incorrect classification)

(1) $$R\left(f\right)=P\left(Y=1,f\left(X\right)<0\right)+P\left(Y=-1,f\left(X\right)\geq 0\right).$$

Let η(x)=P(Y=1|X=x). It is clear that $f_{B}\left(x\right)=\mathrm{sign}\left(2\eta \left(x\right)-1\right)$ minimizes the risk (1) in the family of all classifiers. It is called the Bayes classifier and we denote its risk as $R_{B}=R\left(f_{B}\right).$ Obviously, in practice we do not know the function η, so we cannot find the Bayes classifier. However, if we possess a training sample $\left(X_{1},Y_{1}\right),\ldots ,\left(X_{n},Y_{n}\right)$ containing independent copies of (X,Y), then we can consider a sample analog of (1), namely the empirical misclassification risk

(2) $$\frac{1}{n}\sum_{i=1}^{n}\left[I\left(Y_{i}=1,f\left(X_{i}\right)<0\right)+I\left(Y_{i}=-1,f\left(X_{i}\right)\geq 0\right)\right],$$

where I is the indicator function. Then a minimizer of (2) could be used as our estimator.

The main difficulty in this approach lies in discontinuity of the function (2). It entails that finding its minimizer is computationally difficult and not effective. To overcome this problem, one usually replaces the discontinuous loss function by its convex analog ϕ:R→[0,∞], for instance the logistic loss, the hinge loss or the exponential loss. Then we obtain the convex empirical risk

(3) $$\bar{Q}\left(f\right)=\frac{1}{n}\sum_{i=1}^{n}\phi \left(Y_{i}f\left(X_{i}\right)\right).$$

In the high-dimensional case one usually obtains an estimator by minimizing the penalized version of (3). Those tricks have been successfully used in the classification theory and have allowed to invent boosting algorithms [11], support vector machines [12] or Lasso estimators [3]. In this paper we are mainly interested in Lasso estimators, because they are able to solve both variable selection and prediction problems simultaneously, while the first two algorithms are developed mainly for prediction.

Thus, we consider linear classifiers

(4) $$f_{b}\left(x\right)=b_{0}+\sum_{j=1}^{p}b_{j}x_{j},$$

where $b=\left(b_{0},b_{1},\ldots ,b_{p}\right)\in R^{p+1}.$ For a fixed loss function ϕ we define the Lasso estimator as

(5) $$\hat{b}=\arg\min_{b\in R^{p+1}}\;\bar{Q}\left(f_{b}\right)+\lambda \sum_{j=1}^{p}\left|b_{j}\right|,$$

where λ is a positive tuning parameter, which provides a balance between minimizing the empirical risk and the penalty. The form of the penalty is crucial, because its singularity at the origin implies that some coordinates of the minimizer $\hat{b}$ are exactly equal to zero, if λ is sufficiently large. Thus, calculating (5) we simultaneously select significant predictors in the model and we estimate their coefficients, so we are also able to predict the class of new objects. The function $\bar{Q}\left(f_{b}\right)$ and the penalty are convex, so (5) is a convex minimization problem, which is an important fact from both practical and theoretical points of view. Notice that the intercept $b_{0}$ is not penalized in (5).

The random vector (5) is an estimator of

(6) $$b_{*}=\arg\min_{b\in R^{p+1}}\;Q\left(f_{b}\right),$$

where $Q\left(f_{b}\right)=E\phi \left(Yf_{b}\left(X\right)\right).$ In this paper we are mainly interested in minimizers (6) corresponding to quadratic and logistic loss functions. The latter has a nice information-theoretic interpretation. Namely, it can be viewed as the Kullback–Leibler projection of unknown η on logistic models [13]. The Kullback–Leibler divergence [14] plays an important role in the information theory and statistics, for instance it is involved in information criteria in model selection [15] or in detecting influenctial observations [16].

In general, the classifier corresponding to (6) need not coincide with the Bayes classifier. Obviously, we want to have a “good” estimator, which means that its misclassification risk should be as close to the risk of the Bayes classifier as possible. In other words, its excess risk

(7) $$E\left(\hat{b},f_{B}\right)=E_{D}R\left(\hat{b}\right)-R_{B}$$

should be small, where $E_{D}$ is the expectation with respect to the data $D=\{\left(X_{1},Y_{1}\right),\ldots ,\left(X_{n},Y_{n}\right)\}$ and we write simply R(b) instead of $R(f_{b}).$ Our goal is to study the excess risk (7) for the estimator (5) with different loss functions ϕ. We do it by looking for the upper bounds of (7).

In the excess risk (7) we compare two misclassification risks defined in (1). In the literature one can also find a different approach, which replaces the misclassification risks R(·) in (7) by the convex risks Q(·). In that case the excess risk depends on the loss function ϕ. To deal with this fact one uses the results from [17,18], which state the relation between the excess risk (7) and its analog based on the convex risk Q(·). In this paper we do not follow this way and work, right from the beginning, with the excess risk independent of ϕ. Only the estimator (5) depends on the loss ϕ.

In this paper we are also interested in variable selection. We investigate this problem in the following semiparametric model

(8) $$\eta \left(x\right)=g\left(\beta_{0}+\sum_{j=1}^{p}\beta_{j}x_{j}\right),$$

where η(x)=P(Y=1|X=x), $\beta \in R^{p+1}$ is the true parameter and *g* is unknown function. Thus, we suppose that predictors influence class probability through the function *g* of the linear combination $\beta_{0}+\sum_{j=1}^{p}\beta_{j}x_{j}$. The goal of variable selection is the identification of the set of significant predictors

(9) $$T=\{1\leq j\leq p:\beta_{j}\neq 0\}.$$

Obviously, in the model (8) we cannot estimate an intercept $\beta_{0}$ and we can identify the vector $\left(\beta_{1},\ldots ,\beta_{p}\right)$ only up to a multiplicative constant, because any shift or scale change in $\beta_{0}+\sum_{j=1}^{p}\beta_{j}X_{j}$ can be absorbed by *g*. However, we show in Section 5 that in many situations the Lasso estimator (5) can properly identify the set (9).

The literature on the classification problem is comprehensive. We just mention a few references: [12,19,20,21]. The predictive quality of classifiers is often investigated by obtaining upper bounds for their excess risks. It is an important problem and was studied thoroughly, among others in [17,18,22,23,24]. The variable selection and predictive properties of estimators in the high-dimensional scenario were studied, for instance, in [2,10,13,25,26]. In the current paper we investigate the behaviour of classifiers in possibly misspecified high-dimensional classification, which appears frequently in practice. For instance, while working with binary regression one often assumes incorrectly that the data follow the logistic regression model. Then the problem is solved using the Lasso penalized maximum likelihood method. Another approach to binary regression, which is widely used due to its computational simplicity, is just treating labels $Y_{i}$ as they were numbers and applying standard Lasso. For instance, such method is used in ([1], [Subsections 4.2 and 4.3]) or ([2], Subsection 2.4.1). These two approaches to classification sometimes give unexpectedly good results in variable selection and prediction, but the reason of this phenomenon has not been deeply studied in the literature. Among the above mentioned papers only [2,13,25] take up this issue. However, [25] focuses mainly on the predictive properties of Lasso classifiers with the hinge loss. Bühlmann and van de Geer [2] and Kubkowski and Mielniczuk [13] study general Lipschitz loss functions. The latter paper considers only the variable selection problem. In [2] one also investigates prediction, but they do not study classification with the quadratic loss.

In this paper we are interested in both variable selection and predictive properties of classifiers with convex (but not necessarily Lipschitz) loss functions. The prominent example is classification with the quadratic loss function, which has not been investigated so far in the context of the high-dimensional misspecified model. In this case the estimator (5) can be calculated efficiently using the existing algorithms, for instance [27] or [28], even if the number of predictors is much larger than the sample size. It makes this estimator very attractive, while working with large data sets. In [28] one provides also the efficient algorithm for Lasso estimators with the logistic loss in the high-dimensional scenario. Therefore, misspecified classification with the logistic loss plays an important role in this paper as well. Our goal is to study thoroughly such estimators and provide conditions, which guarantee that they are successful in prediction and variable selection.

The paper is organized as follows: in the next section we provide basic notations and assumptions, which are used in this paper. In Section 3 we study predictive properties of Lasso estimators with different loss functions. We will see that these properties depend strongly on the estimation quality of estimators, which is studied in Section 4. In Section 5 we consider variable selection. In Section 6 we show numerical experiments, which describe the quality of estimators in practice. The proofs and auxiliary results are relegated to Appendix A.

## 2. Assumptions and Notation

In this paper we work in the high-dimensional scenario p>>n. As usual we assume that the number of predictors *p* can vary with the sample size n, which could be denoted as $p\left(n\right)=p_{n}.$ However, to make notation simpler we omit the lower index and write *p* istead of $p_{n}.$ The same refers to the other objects appearing in this paper.

In the further sections we will need the following notation:

- $X_{i}={\left(X_{i1},X_{i2},\ldots ,X_{ip}\right)}^{⊤}$;
- $X={\left(X_{1},X_{2},\ldots ,X_{n}\right)}^{⊤}$ is the (n×p)-matrix of predictors;
- Let A⊂{1,…,p}. Then $A^{c}=\left\{1,\ldots ,p\right\}\backslash A$ is a complement of *A*;
- $X_{A}$ is a submatrix of X, with columns whose indices belong to *A*;
- $b_{A}$ is a restriction of a vector $b\in R^{p}$ to the indices from A;
- |A| is the number of elements in A;
- $\tilde{A}=A\cup \left\{0\right\},$ so the set $\tilde{A}$ contains indices from *A* and the intercept;
- The $l_{q}$-norm of a vector is defined as ${\left|b\right|}_{q}={\left(\sum_{j=1}^{p}{\left|b_{j}\right|}^{q}\right)}^{1/q}$ for q∈[1,∞];
- For $x\in R^{p}$ we denote $\tilde{x}={\left(1,x\right)}^{⊤};$
- $\tilde{X}$ is the matrix X with the column of ones binded from the left side;
- ${\hat{b}}^{\;quad},b_{*}^{quad}$ are minimizers in (5), (6), respectively, with the quadratic loss function;
- ${\hat{b}}^{log},b_{*}^{log}$ are minimizers in (5), (6), respectively, with the logistic loss function;
- The Kullback–Leibler (KL) distance [14] between two binary distributions with success probabilities $\pi_{1}$ and $\pi_{2}$ is defined as
  (10) $$KL\left(\pi_{1},\pi_{2}\right)=\pi_{1}\log\left(\frac{\pi_{1}}{\pi_{2}}\right)+\left(1-\pi_{1}\right)\log\left(\frac{1-\pi_{1}}{1-\pi_{2}}\right)\;.$$

Obviously, we have $KL(\pi_{1},\pi_{2})\geq 0$ and $KL(\pi_{1},\pi_{2})=0$ if only if $\pi_{1}=\pi_{2}$. Moreover, the KL distance need not be symmetric;

- the set of nonzero coefficients of $b_{*}^{quad}$ is denoted as
  (11) $$T=\{1\leq j\leq p:{\left(b_{*}^{\;quad}\right)}_{j}\neq 0\}.$$

Notice that the intercept is not contained in (11) even if it is nonzero.

We also specify assumptions, which are used in this paper.

**Assumption** **1.** *We assume that $\left(X,Y\right),\left(X_{1},Y_{1}\right),\ldots ,\left(X_{n},Y_{n}\right)$ are i.i.d. random vectors. Moreover, predictors are univariate subgaussian, i.e., for each a∈R and j∈{1,…,p} we have $E\exp\left(aX_{j}\right)\leq \exp\left(\sigma_{j}^{2}a^{2}/2\right)$ for positive numbers $\sigma_{j}.$ We also denote $\sigma =\max_{1\leq j\leq p}\sigma_{j}.$ Finally, we suppose that the matrix $H=E[XX^{⊤}]$ is positive definite and $H_{jj}=1$ for j=1,…,p.*

In Section 4 and Section 5 we need stronger version of Assumption 1.

**Assumption** **2.** *We suppose that the subvector of predictors $X_{T}$ is subgaussian with the coefficient $\sigma_{0}>0$, i.e., for each $u\in R^{\left|T\right|}$ we have $E\exp\left(u^{⊤}X_{T}\right)\leq \exp\left(\sigma_{0}^{2}u^{⊤}H_{T}u/2\right),$ where $H_{T}={\left(E[X_{1j}X_{1k}]\right)}_{j,k\in T}.$ The remaining conditions are as in Assumption 1. We also denote $\sigma =\max(\sigma_{0},\sigma_{j},j\notin T).$*

Subgaussianity of predictors is a standard assumption while working with random predictors in high-dimensional models, cf. [13]. In particular, Assumption 1 implies that E[X]=0 and σ≥1 [29].

## 3. Predictive Properties of Classifiers

In this part of the paper we study prediction properties of classifiers with convex loss functions. To do it we look for upper bounds of the excess risk (7) of estimators.

As usual the excess risk in (7) can be decomposed as

(12) $$E_{D}R\left(\hat{b}\right)-R\left(b_{*}\right)\;+\;R\left(b_{*}\right)-R_{B}.$$

The second term in (12) is the approximation risk and compares the predictive abilitity of the “best” linear classifier (6) to the Bayes classifier. The first term in (12) is called the estimation risk and describes how the estimation process influences the predictive property of classifiers.

In the next theorem we bound from above the estimation risk of classifiers. To make the result more transparent we use notations $P_{D}$ and $P_{X}$ in (13), which indicate explicitly which probability we consider, i.e., $P_{D}$ is probability with respect to the data *D* and $P_{X}$ is with respect to the new object *X*. In further results we omit these lower indexes and believe that it does not lead to confusion.

**Theorem** **1.** For c>0 we consider an event $\Omega =\{|\hat{b}-b_{*}{|}_{1}\leq c\}.$ We have

(13) $$E_{D}R\left(\hat{b}\right)-R\left(b_{*}\right)\leq 2P_{D}\left(\Omega^{c}\right)+P_{X}\left(\right|b_{*}^{⊤}\tilde{X}\left|\leq c\right|\tilde{X}{|}_{\infty }).$$

In Theorem 1 we obtain the upper bound for the estimation risk. This risk becomes small, if we establish that probability of the event $\Omega^{c}$ is small and the sequence c, which is involved in Ω and in the second term on the right-hand side of (13), decreases sufficiently fast to zero. Therefore, Theorem 1 shows that to have a small estimation risk it is enough to prove that for each ε∈(0,1) there exists *c* such that

(14) $$P(|\hat{b}-b_{*}{|}_{1}\leq c)\geq 1-\varepsilon .$$

Moreover, numbers ε and *c* should be sufficiently small. This property will be studied thoroughly in the next section. Notice that the first term on the right-hand side of (13) relates to the fact, how well (5) estimates (6). Moreover, the second expression on the right-hand side of (13) can be bounded from above, if predictors are sufficiently regular, for instance subgaussian.

So far, we have been interested in the estimation risk of estimators. In the next result we establish the upper bound for the approximation risk as well. This bound combined with (13) enables us to bound from above the excess risk of estimators. We prove this fact for the quadratic loss $\phi \left(t\right)={\left(1-t\right)}^{2}$ and the logistic loss $\phi \left(t\right)=\log\left(1+e^{-v}\right)$, which play prominent roles in this paper.

**Theorem** **2.** Suppose that Assumption 1 is fulfilled. Moreover, a random variable $b_{*}^{⊤}\tilde{X}$ has a density h, which is continuous on the interval $U=[-2\sigma c\sqrt{\log p},2\sigma c\sqrt{\log p}\;]$ and $\tilde{h}=\underset{u\in U}{\sup}h\left(u\right).$

*(a) We have*

(15) $$\begin{array}{ccc} E({\hat{b}}^{quad},f_{B}) & \leq & 2P\left(\Omega^{c}\right)+4\sigma {\tilde{h}}^{quad}c\sqrt{\log p}+2/p \end{array}$$

(16) $$\begin{array}{cc} + & \sqrt{E{\left[2\eta \left(X\right)-1-{\left(b_{*}^{quad}\right)}^{⊤}\tilde{X}\right]}^{2}}\;, \end{array}$$

where ${\tilde{h}}^{quad}$ refers to the density h of ${\left(b_{*}^{quad}\right)}^{⊤}\tilde{X}.$

*(b) Let $\eta_{log}\left(u\right)=1/\left(1+\exp\left(-u\right)\right).$ Then we obtain*

(17) $$\begin{array}{ccc} E({\hat{b}}^{log},f_{B}) & \leq & 2P\left(\Omega^{c}\right)+4\sigma {\tilde{h}}^{log}c\sqrt{\log p}+2/p \end{array}$$

(18) $$\begin{array}{cc} + & \sqrt{2E\left[KL\left(\eta \left(X\right),\eta_{log}\left({\left(b_{*}^{log}\right)}^{⊤}\tilde{X}\right)\right)\right]} \end{array}$$

where KL(·,·) is the Kullback–Leibler distance defined in (10) and ${\tilde{h}}^{log}$ refers to the density h of ${\left(b_{*}^{log}\right)}^{⊤}\tilde{X}.$ Additionally, assuming that there exists δ∈(0,1) such that δ≤η(X)≤1−δ and $\delta \leq \eta_{log}\left({\left(b_{*}^{log}\right)}^{⊤}\tilde{X}\right)\leq 1-\delta ,$ we have

(19) $$E\left[KL\left(\eta \left(X\right),\eta_{log}\left({\left(b_{*}^{log}\right)}^{⊤}\tilde{X}\right)\right)\right]\leq {\left(2\delta \left(1-\delta \right)\right)}^{-1}E{\left[\eta \left(X\right)-\eta_{log}\left({\left(b_{*}^{log}\right)}^{⊤}\tilde{X}\right)\right]}^{2}.$$

In Theorem 2 we establish upper bounds on the excess risks for Lasso estimators (5). They describe predictive properties of these classifiers. In this paper we consider linear classifiers, so the misclassification risk of an estimator is close to the Bayes risk, if the “truth” can be approximated linearly in a satisfactory way. For the classifier with the logistic loss this fact is described by (18) and (19), which measure the distance between true success probability and the one in logistic regression. In particular, when the true model is logistic, then (18) and (19) vanish. The expression (16) relates to the approximation error in the case of the quadratic loss. It measures how well the conditional expectation E[Y|X] can be described by the “best” (with respect to the loss ϕ) linear function ${\left(b_{*}^{quad}\right)}^{⊤}\tilde{X}.$

The right-hand sides of (15) and (17) relate to estimation risk. They have been already discussed after Theorem 1. Using subgaussianity of predictors we have made them more explicit. The main ingredient of bounds in Theorem 2, namely $P(\Omega^{c}),$ is studied in the next section.

Results in Theorem 2 refer to Lasso estimators with quadratic and logistic loss functions. Similar results are given in ([2], Theorem 6.4). They refer to the case that the convex excess risk is considered, i.e., the misclassification risks R(·) are replaced by the convex risks Q(·) in (7). Moreover, these results do not consider Lasso estimators with the quadratic loss applied to classification, which is an approach playing a key role in the current paper. Furthermore, in ([2], Theorem 6.4) the estimation error $\hat{b}-b_{*}$ is measured in the $l_{1}$-norm, which is enough for prediction. However, for variable selection the $l_{\infty }$-norm gives better results. Such results will be established in Section 4 and Section 5. Finally, results of [2] need more restrictive assumptions than ours. For instance, predictors should be bounded and a function $f_{b_{*}}$ should be sufficiently close to $f_{B}$ in the supremum norm.

Analogous bounds to those in Theorem 2 can be obtained for other loss functions, if we combine Theorem 1 with results of [17]. Finally, we should stress that the estimator $\hat{b}$ need not rely on the Lasso method. All we require is that the bound (14) can be estiblished for this estimator.

## 4. On the Event Ω

In this section we show that probability of the event Ω can be close to one. Such results for classification models with Lipschitz loss functions were established in [2,13]. Therefore, we focus on the quadratic loss function, which is obviously non-Lipschitz. This loss function is important from the practical point of view, but was not considered in these papers. Moreover, in our results the estimation error in Ω can be measured in the $l_{q}$-norms, q≥1, not only in the $l_{1}$-norm as in [2,13]. Bounds in the $l_{\infty }$-norm lead to better results in variable selection, which are given in Section 5.

We start with introducing the cone invertibility factor (CIF), which plays a significant role in investigating properties of estimators based on the Lasso penalty [9]. In the case n>p one usually uses the minimal eigenvalue of the matrix $X^{⊤}X/n$ to express the strength of correlations between predictors. Obviously, in the high-dimensional scenario this value is equal to zero and the minimal eigenvalue needs to be replaced by some other measure of predictors interdependency, which would describe the potential of consistent estimation of model parameters.

For ξ>1 we define a cone

$$C\left(\xi \right)=\{b\in R^{p+1}:|b_{T^{c}}{|}_{1}\leq \xi |b_{\tilde{T}}{|}_{1}\},$$

where we recall that $\tilde{T}=T\cup \left\{0\right\}.$ In the case when p>>n three different characteristics measuring the potential for consistent estimation of the model parameters have been introduced:

- The restricted eigenvalue [8]:
  $$RE\left(\xi \right)=\underset{0\neq b\in C(\xi )}{\inf}\frac{b^{⊤}{\tilde{X}}^{⊤}\tilde{X}b/n}{{\left|b\right|}_{2}^{2}}\;\;,$$
- The compatibility factor [7]:
  $$K\left(\xi \right)=\underset{0\neq b\in C(\xi )}{\inf}\frac{\left|T\right|b^{⊤}{\tilde{X}}^{⊤}\tilde{X}b/n}{|b_{T}{|}_{1}^{2}}\;\;,$$
- The cone invertibility factor (CIF, [9]): for q≥1
  $${\bar{F}}_{q}\left(\xi \right)=\underset{0\neq b\in C(\xi )}{\inf}\frac{{\left|T\right|}^{1/q}{\left|{\tilde{X}}^{⊤}\tilde{X}b/n\right|}_{\infty }}{{\left|b\right|}_{q}}\;\;.$$

In this article we will use CIF, because this factor allows for a sharp formulation of convergency results for all $l_{q}$ norms with q≥1, see ([9], Section 3.2). The population (non-random) version of CIF is given by

$$F_{q}\left(\xi \right)=\underset{0\neq b\in C(\xi )}{\inf}\frac{{\left|T\right|}^{1/q}{\left|\tilde{H}b\right|}_{\infty }}{{\left|b\right|}_{q}}\;,$$

where $\tilde{H}=E\left[\tilde{X}{\tilde{X}}^{⊤}\right].$ The key property of the random and the population versions of CIF, ${\bar{F}}_{q}\left(\xi \right)$ and $F_{q}\left(\xi \right)$, is that, in contrast to the smallest eigenvalues of matrices ${\tilde{X}}^{⊤}\tilde{X}/n$ and $\tilde{H},$ they can be close to each other in the high-dimensional setting, see ([30], Lemma 4.1) or ([31], Corollary 10.1). This fact is used in the proof of Theorem 3 (given below).

Next, we state the main results of this section.

**Theorem** **3.** *Let a∈(0,1),q≥1 and ξ>1 be arbitrary. Suppose that Assumption 2 is satisfied and*

(20) $$n\geq \frac{K_{1}{\left|T\right|}^{2}\sigma^{4}{\left(1+\xi \right)}^{2}\log\left(p/a\right)}{F_{q}^{2}\left(\xi \right)}$$

*and*

(21) $$\lambda \geq K_{2}\frac{\xi +1}{\xi -1}\sigma^{2}\sqrt{\frac{\log(p/a)}{n}}\;,$$

*where $K_{1},K_{2}$ are universal constants. Then there exists a universal constant $K_{3}>0$ such that with probability at least $1-K_{3}a$ we have*

(22) $$|{\hat{b}}^{quad}-b_{*}^{quad}{|}_{q}\leq \frac{{2\xi |T|}^{1/q}\lambda }{\left(\xi +1\right)F_{q}\left(\xi \right)}\;.$$

In Theorem 3 we provide the upper bound for the estimation error of the Lasso estimator with the quadratic loss function. This result gives the conditions for estimation consistency of ${\hat{b}}^{quad}$ in the high-dimensional scenario, i.e., the number of predictors can be significantly greater than the sample size. Indeed, consistency in the $l_{\infty }$-norm holds e.g., when $p=\exp\left(n^{a_{1}}\right),\left|T\right|=n^{a_{2}},a=\exp\left(-n^{a_{1}}\right),$ where $a_{1}+2a_{2}<1.$ Moreover, λ is taken as the right-hand side of the inequality (21) and finally $F_{\infty }\left(\xi \right)$ is bounded from below (or slowly converging to 0) and σ is bounded from above (or slowly diverging to *∞*).

The choice of the λ parameter is difficult in practice, which is a common drawback of Lasso estimators. However, Theorem 3 gives us a hint how to choose λ. The “safe” choice of λ is the right-hand side of the inequality (21), so, roughly speaking, λ should be proportional to $\sqrt{\log(p)/n}.$ In the experimental part of the paper the parameter λ is chosen using the cross-validation method. As we will observe it gives satisfatory results for the Lasso estimators in both prediction and variable selection.

Theorem 3 is a crucial fact, which gives the upper bound for (15) in Theorem 2. Namely, taking q=1,a=1/p and λ equal to the right-hand side of the inequality (21), we obtain the following consequence of Theorem 3.

**Corollary** **1.** *Suppose that Assumptions 2 is satisfied. Moreover, assume that there exist $\xi_{0}>1$ and constants $C_{1}>0$ and $C_{2}<\infty$ such that $F_{1}\left(\xi_{0}\right)\geq C_{1}$ and $\sigma \leq C_{2}$. If $n\geq K_{1}{\left|T\right|}^{2}\log p,$ then*

(23) $$P\left(|{\hat{b}}^{quad}-b_{*}^{quad}{|}_{1}\leq K_{2}\left|T\right|\sqrt{\frac{\log p}{n}}\right)\geq 1-K_{3}/p,$$

*where the constants $K_{1}$ and $K_{2}$ depend only on $\xi_{0},C_{1},C_{2}$ and $K_{3}$ is a universal constant provided in Theorem 3.*

The above result works for Lasso estimators with the quadratic loss. In the case of the logistic loss analogous result is obtained in ([13], Theorem 1). In fact, their results relate to the case of quite general Lipschitz loss functions, which can be useful in extending Theorem 2 to such cases.

## 5. Variable Selection Properties of Estimators

In Section 3 we are interested in predictive properties of estimators. In this part of the paper we focus on variable selection, which is another important problem in high-dimensional statistics. As we have already noticed upper bounds for probability of the event Ω are crucial in proving results concerning prediction. It also plays a key role in establishing results relating to variable selection. In this section we again focus on the Lasso estimators with the quadratic loss functions. The analogous results for Lipschitz loss functions were considered in ([13], Corollary 1).

In the variable selection problem we want to find significant predictors, which, roughly speaking, give us some information on the observed phenomenon. We consider this problem in the semiparametric model, which is defined in (8). In this case the set of significant predictors is given by (9). As we have already mentioned vectors β and $b_{*}^{quad}$ need not be the same. However, in [32] one proved that for a real number γ the following relation

(24) $${\left(b_{*}^{quad}\right)}_{j}=\gamma \beta_{j},\;j=1,\ldots ,p$$

holds under Assumption 3, which is now stated.

**Assumption** **3.** *Let $\overset{˚}{\beta }=\left(\beta_{1},\ldots ,\beta_{p}\right).$ We assume that for each $\theta \in R^{p}$ the conditional expectation $E\left[\theta^{⊤}X|{\overset{˚}{\beta }}^{⊤}X\right]$ exists and*

$$E\left[\theta^{⊤}X|{\overset{˚}{\beta }}^{⊤}X\right]=d_{\theta }{\overset{˚}{\beta }}^{⊤}X$$

*for a real number $d_{\theta }\in R.$*

The coefficient γ in (24) can be easily calculated. Namely, we have

$$\gamma =\frac{E\left[Y{\overset{˚}{\beta }}^{⊤}X\right]}{{\overset{˚}{\beta }}^{⊤}H\overset{˚}{\beta }}=\frac{2E\left[g\left(\beta^{⊤}\tilde{X}\right){\overset{˚}{\beta }}^{⊤}X\right]}{{\overset{˚}{\beta }}^{⊤}H\overset{˚}{\beta }}\;.$$

Standard arguments [33] show that γ is nonzero, if *g* is monotonic. In this case we have that the set T defined in (9) equals to *T* defined in (11).

Assumption 3 is a well-known condition in the literature, see e.g., [13,32,34,35,36]. It is always satisfied in the simple regression model (i.e., when $X_{1}\in R$), which is often used for initial screening of explanatory variables, see, e.g., [37]. It is also satisfied when *X* comes from the *elliptical distribution*, like the multivariate normal distribution or multivariate *t*-distribution. In the interesting paper [38] one advocates that Assumption 3 is a nonrestrictive condition, when the number of predictors is large, which is the case that we focus on in this paper.

Now, we state the results of this part of the paper. We will use the notation $b_{min}^{quad}=\min_{j\in T}\left|{\left(b_{*}^{quad}\right)}_{j}\right|.$

**Corollary** **2.** *Suppose that conditions of Theorem 3 are satisfied for q=∞. If $b_{min}^{quad}\geq \frac{4\xi \lambda }{\left(\xi +1\right)F_{\infty }\left(\xi \right)},$ then*

$$P\left(\forall_{j\in T,k\notin T}\;|{\hat{b}}_{j}^{quad}\left|>\right|{\hat{b}}_{k}^{quad}|\right)\geq 1-K_{3}a\;,$$

*where $K_{3}$ is the universal constant from Theorem 3.*

In Corollary 2 we show that the Lasso estimator with the quadratic loss is able to separate predictors, if the nonzero coefficients of ${\overset{˚}{b}}_{*}^{quad}$ are large enough in absolute values. In the case that *T* equals (9) (i.e., *T* is the set of significant predictors) we can prove that the thresholded Lasso estimator is able to find the true model with high-probability. This fact is stated in the next result. The thresholded Lasso estimator is denoted by ${\hat{b}}_{th}^{quad}$ and defined as

(25) $${\left({\hat{b}}_{th}^{quad}\right)}_{j}={\hat{b}}_{j}^{quad}I(|{\hat{b}}_{j}^{quad}\left|\geq \delta \right),\;j=1,\ldots ,p,$$

where δ>0 is a threshold. We set ${\left({\hat{b}}_{th}^{quad}\right)}_{0}={\hat{b}}_{0}^{quad}$ and denote ${\hat{T}}_{th}=\left\{1\leq j\leq p:{\left({\hat{b}}_{th}^{quad}\right)}_{j}\neq 0\right\}.$

**Corollary** **3.** *Let g in (8) be monotonic. We suppose that Assumption 3 and conditions of Theorem 3 are satisfied for q=∞. If*

$$\frac{2\xi \lambda }{\left(\xi +1\right)F_{\infty }\left(\xi \right)}<\delta \leq b_{min}^{quad}/2,$$

*then*

$$P\left({\hat{T}}_{th}=T\right)\geq 1-K_{3}a,$$

*where $K_{3}$ is the universal constant from Theorem 3.*

Corollary 3 states that the Lasso estimator after thresholding is able to find the true model with high probability, if the threshold is appropriately chosen. However, Corollary 3 does not give a constructive way of choosing the threshold, because both endpoints of the interval $\left[\frac{2\xi \lambda }{\left(\xi +1\right)F_{\infty }\left(\xi \right)},b_{min}^{quad}/2\right]$ are unknown. It is not a surprising fact and has been already observed, for instance, in linear models ([9], Theorem 8). In the literature we can find methods, which help to choose a threshold in practice, for instance the approach relying on information criteria developed in [39,40].

Finally, we discuss the condition of Corollary 3 that $b_{min}^{quad}$ cannot be too small, i.e., $b_{min}^{quad}\geq \frac{4\xi \lambda }{\left(\xi +1\right)F_{\infty }\left(\xi \right)}$. We know that ${\left(b_{*}^{quad}\right)}_{j}=\gamma \beta_{j}$ for j=1,…,p, so the considered condition requires that

(26) $$\min_{j\in T}\left|\beta_{j}\right|\geq \frac{4\xi \lambda }{|\gamma |\left(\xi +1\right)F_{\infty }\left(\xi \right)}\;.$$

Compared to the similar condition for the Lasso estimators in the well-specified models, we observe that the denominator in (26) contains an additional factor |γ|. This number is usually smaller than one, which means that in the misspecified models the Lasso estimator needs larger sample size to work well. This phenomenon is typical for misspecified models and the similar restrictions hold for competitors [13].

## 6. Numerical Experiments

In this section we present simulation study, where we compare the accuracy of considered estimators in prediction and variable selection.

We consider the model (8) with predictors generated from the *p*-dimensional normal distribution N(0,H), where $H_{jj}=1$ and $H_{jk}=0.5$ for j≠k. The true parameter is

(27) $$\beta =(1,\underset{10}{\underset{︸}{\pm 1,\pm 1,\ldots ,\pm 1}},0,0,\ldots ,0),$$

where signs are chosen at random. The first coordinate in (27) corresponds to the intercept and the next ten coefficients relate to significant predictors in the model. We study two cases:

- Scenario 1: g(x)=exp(x)/(1+exp(x));
- Scenario 2: g(x)=arctan(x)/π+0.5.

In each scenario we generate the data $\left(X_{1},Y_{1}\right),\ldots ,\left(X_{n},Y_{n}\right)$ for n∈{100,350,600}. The corresponding numbers of predictors are p∈{100,1225,3600}, so the number of predictors exceeds significantly the sample size in the experiments. For every model we consider two Lasso estimators with unpenalized intercepts (5): the first one with the logistic loss and the second one with the quadratic loss. They are denoted by “logistic” and “quadratic”, respectively. To calculate them we use the “glmnet” package [28] in the “R” software [41]. The tuning parameters λ are chosen on the basis of 10-fold cross-validation.

Observe that applying the Lasso estimator with the logistic loss function to Scenario 1 leads to a well-specified model, while using the quadratic loss implies misspecification. In Scenario 2 both estimators work in misspecified models.

Simulations for each scenario are repeated 300 times.

To describe the quality of estimators in variable selection we calculate two values:

- **TD**—the number of correctly selected relevant predictors;
- **sep**—the number of relevant predictors, whose Lasso coefficients are greater in absolute value than the largest in absolute value Lasso coefficient corresponding to irrelevant predictors.

So, we want to confirm that the considered estimators are able to separate predictors, which we establish in Section 5. Using TD we also study “screening” properties of estimators, which are easier than separability.

The classification accuracy of estimators is measured in the following way: we generate a test sample containg 1000 objects. On this set we calculate

- **pred**—the fraction of correctly predicted classes of objects for each estimator.

The results of experiments are collected in Table 1 and Table 2. By the “oracle” we mean the classifier, which works only with significant predictors and uses the function *g* from the true model (8) in the estimation process.

Finally, we also compare execution time of both algorithms. In Table 3 we show the averaged relative time difference

(28) $$\frac{t^{log}-t^{quad}}{t^{quad}}\;,$$

where $t^{quad}$ and $t^{log}$ is time of calculating Lasso with quadratic and logistic loss functions, respectively.

Looking at the results of experiments we observe that both estimators perform in a satisfactory way. Their predictive accuracy is relatively close to the oracle, especially when the sample size is larger. In variable selection we see that both estimators are able to find significant predictors and separate predictors in both scenarios. Again we can notice that properties of estimators become better, when *n* increases.

In Scenario 2 the quality of both estimators in prediction and variable selection is comparable. In Scenario 1, which is well-specified for Lasso with the logistic loss, we observe its dominance over Lasso with the quadratic loss. However, this dominance is not large. Therefore, using Lasso with the quadratic loss we obtain slightly worse accuracy of the procedure, but this algorithm is computationally faster. The computational efficiency is especially important, when we study large data sets. As we can see in Table 3 execution time of estimators is almost the same for n=350, but for n=600 the relative time difference becomes greater than 10%.

## Figures and Tables

**Table 1:** Results for Scenario 1.

n=100	Quadratic	Logistic	Oracle
TD	6.3	6.1	
sep	2.2	2.3	
pred	0.734	0.736	0.810
n=350			
TD	9.3	9.5	
sep	6.0	6.3	
pred	0.774	0.779	0.831
n=600			
TD	9.8	9.9	
sep	8.6	8.9	
pred	0.791	0.795	0.832

**Table 2:** Results for Scenario 2.

n=100	Quadratic	Logistic	Oracle
TD	4.8	4.6	
sep	1.4	1.4	
pred	0.697	0.698	0.768
n=350			
TD	8.1	8.2	
sep	3.9	3.9	
pred	0.730	0.731	0.805
n=600			
TD	9.4	9.4	
sep	6.8	6.9	
pred	0.750	0.752	0.809

**Table 3:** Relative time difference (28) of algorithms.

	Scenario 1	Scenario 2
n=350	0.02	0.06
n=600	0.11	0.13

## Appendix A. Proofs and Auxiliary Results

This section contains proofs of results from the paper. Additional lemmas are also provided.

### Appendix A.1. Results from Section 3

**Proof of Theorem** **1.** For arbitrary $b\in R^{p+1}$ the averaged misclassification risk of $f_{b}$ can be expressed as

(A1) $$E_{D}R\left(b\right)=E_{D}E_{\left(X,Y\right)}\left[I\left(Y=1\right)I\left(b^{⊤}\tilde{X}<0\right)+I\left(Y=-1\right)I\left(b^{⊤}\tilde{X}\geq 0\right)\right].$$

Moreover, we have

(A2) $$I\left(Y=-1\right)I\left(b^{⊤}\tilde{X}\geq 0\right)=I\left(Y=-1\right)\left[1-I(b^{⊤}\tilde{X}<0)\right].$$

Applying (A1) and (A2) for $\hat{b}$ and $b_{*},$ we obtain

$$\begin{array}{cc} \; & |E_{D}R\left(\hat{b}\right)-R\left(b_{*}\right)|\\ = & \left|E_{D}E_{\left(X,Y\right)}\left[I(Y=1)-I(Y=-1)\right]\left[I\left({\hat{b}}^{⊤}\tilde{X}<0\right)-I\left(b_{*}^{⊤}\tilde{X}<0\right)\right]\right|\\ \leq & E_{D}E_{\left(X,Y\right)}\left|I\left({\hat{b}}^{⊤}\tilde{X}<0\right)-I\left(b_{*}^{⊤}\tilde{X}<0\right)\right|\\ = & P\left({\hat{b}}^{⊤}\tilde{X}<0,b_{*}^{⊤}\tilde{X}\geq 0\right)+P\left({\hat{b}}^{⊤}\tilde{X}\geq 0,b_{*}^{⊤}\tilde{X}<0\right), \end{array}$$

where *P* is probability with respect to both the data *D* and the new object X. Observe that on the event Ω, we have that

$${\hat{b}}^{⊤}\tilde{X}\leq c{\left|\tilde{X}\right|}_{\infty }+b_{*}^{⊤}\tilde{X},$$

so

$$\begin{array}{cc} \; & P\left({\hat{b}}^{⊤}\tilde{X}\geq 0,b_{*}^{⊤}\tilde{X}<0\right)=P\left({\hat{b}}^{⊤}\tilde{X}\geq 0,b_{*}^{⊤}\tilde{X}<0,\Omega \right)\\ + & P({\hat{b}}^{⊤}\tilde{X}\geq 0,b_{*}^{⊤}\tilde{X}<0,\Omega^{c})\\ \leq & P_{X}\left(-c\right|\tilde{X}{|}_{\infty }\leq b_{*}^{⊤}\tilde{X}<0)+P_{D}\left(\Omega^{c}\right). \end{array}$$

Analogously, we obtain

$$P\left({\hat{b}}^{⊤}\tilde{X}<0,b_{*}^{⊤}\tilde{X}\geq 0\right)\leq P_{X}(0\leq b_{*}^{⊤}\tilde{X}\leq c|\tilde{X}{|}_{\infty })+P_{D}\left(\Omega^{c}\right)$$

from

$${\hat{b}}^{⊤}\tilde{X}\geq -c{\left|\tilde{X}\right|}_{\infty }+b_{*}^{⊤}\tilde{X},$$

which finishes the proof. □

**Lemma** **A1.**
*Suppose that Assumption 1 is fulfilled. Moreover, a random variable $b_{*}^{⊤}\tilde{X}$ has a density h, which is continuous on the interval $U=[-2\sigma c\sqrt{\log p},2\sigma c\sqrt{\log p}\;]$ and $\tilde{h}=\underset{u\in U}{\sup}h\left(u\right).$ Then*

(A3) $$P_{X}\left(\right|b_{*}^{⊤}\tilde{X}\left|\leq c\right|\tilde{X}{|}_{\infty })\leq 4\sigma \tilde{h}c\sqrt{\log p}+2/p.$$

**Proof.** For simplicity, we omit the lower index *X* in probability $P_{X}$ in this proof. We take a>1 and obtain inequalities

(A4) $$\begin{array}{cc} & P(|b_{*}^{⊤}\tilde{X}\left|\leq c\right|\tilde{X}{|}_{\infty })\leq P(|b_{*}^{⊤}\tilde{X}\left|\leq c\right|\tilde{X}{|}_{\infty },|\tilde{X}{|}_{\infty }\leq a)\\ + & P(|b_{*}^{⊤}\tilde{X}|\leq c|\tilde{X}{|}_{\infty },|\tilde{X}{|}_{\infty }>a)\\ \leq & P(|b_{*}^{⊤}\tilde{X}\left|\leq ca)+P(\right|\tilde{X}{|}_{\infty }>a). \end{array}$$

The second expression in (A4) equals $P(|X{|}_{\infty }>a),$ because a>1. It can be handled using subgaussianity of *X* as follows: take z>0 and notice that by the Markov inequality and the fact that exp(|u|)≤exp(u)+exp(−u) for each u∈R, we obtain

$$\begin{array}{ccc} P(|X{|}_{\infty }>a) & \leq & e^{-za}{E\exp(z|X|}_{\infty })\leq e^{-za}\sum_{j=1}^{p}E\exp(z|X_{j}\left|\right)\\ & \leq & 2p\exp(\sigma^{2}z^{2}/2-az). \end{array}$$

Taking $z=a/\sigma^{2},$ we obtain

$${P(|X|}_{\infty }>a)\leq 2p\exp\left(-a^{2}/\left(2\sigma^{2}\right)\right).$$

Then we choose $a=2\sigma \sqrt{\log p},$ which is not smaller than one, because σ≥1 from Assumption 1. Finally, the first term in (A4) can be bounded from above by $2ca\tilde{h}=4\sigma \tilde{h}c\sqrt{\log p}$ by the mean value theorem. □

**Proof of Theorem** **2.** The right-hand side of (15) and (17) are upper bounds on the estimation risk. They are obtained using Theorem 1 and Lemma A1. The expressions (16) and (18) are upper bounds for the approximation risk in the case of estimators with the quadratic and logistic loss functions, respectively. In particular, (16) follows from ([17], [Theorem 2.1) applied for $f_{b_{*}^{quad}}$ and Example 3.1. Establishing (18) is similar: we just use ([17], [Theorem 2.1) applied for $f_{b_{*}^{log}}$ and Example 3.5 to show that

(A5) $$R\left(b_{*}^{log}\right)-R_{B}\leq \sqrt{2E\left[KL\left(\eta \left(X\right),\eta_{log}\left({\left(b_{*}^{log}\right)}^{⊤}\tilde{X}\right)\right)\right]},$$

where the Kullback–Leibler distance KL(·,·) is defined in (10). Next, we define the function h(a)=aloga+(1−a)log(1−a) for a∈(0,1). Clearly, we have $KL\left(a,b\right)=h\left(a\right)-h\left(b\right)-h^{\prime }\left(b\right)\left(a-b\right)$ and $h^{\prime \prime }\left(a\right)={\left(a\left(1-a\right)\right)}^{-1.}$ Therefore, from the mean value theorem

(A6) $$KL\left(a,b\right)=\frac{{\left(a-b\right)}^{2}}{2c(1-c)}$$

for some *c* between *a* and *b*. To finish the proof we apply (A6) to the right-hand side of (A5) with δ<c<1−δ. □

### Appendix A.2. Results from Section 4

To simplify notation we write $\hat{b},b_{*}$ for ${\hat{b}}^{quad},b_{*}^{quad},$ respectively, in this section. Moreover, we also denote ${\overset{˚}{b}}_{*}=\left({\left(b_{*}\right)}_{1},\ldots ,{\left(b_{*}\right)}_{p}\right).$

We start with establishing results, which help us to prove Theorem 3.

**Lemma** **A2.**
*For ${\overset{˚}{b}}_{*}=H^{-1}E\left[XY\right]$ we have ${\overset{˚}{b}}_{*}^{⊤}H{\overset{˚}{b}}_{*}\leq 1.$*

**Proof.** The proof is elementary and based on the inequality

(A7) $$0\leq E{\left[E\left[Y|X\right]-{\overset{˚}{b}}_{*}^{⊤}X\right]}^{2}.$$

The right-hand side of (A7) can be expressed as

(A8) $$E\left[{\left[E\left[Y|X\right]\right]}^{2}-2{\overset{˚}{b}}_{*}^{⊤}E\left[XY|X\right]+{\overset{˚}{b}}_{*}^{⊤}XX^{⊤}{\overset{˚}{b}}_{*}\right]=E{\left[E\left[Y|X\right]\right]}^{2}-2{\overset{˚}{b}}_{*}^{⊤}E\left[XY\right]+{\overset{˚}{b}}_{*}^{⊤}H{\overset{˚}{b}}_{*}.$$

Using ${\overset{˚}{b}}_{*}=H^{-1}E\left[XY\right],$ we have ${\overset{˚}{b}}_{*}^{⊤}E\left[XY\right]={\overset{˚}{b}}_{*}^{⊤}HH^{-1}E\left[XY\right]={\overset{˚}{b}}_{*}^{⊤}H{\overset{˚}{b}}_{*}$ and we can bound from above the right-hand side of (A8) by

$$E\left[Y^{2}\right]-{\overset{˚}{b}}_{*}^{⊤}H{\overset{˚}{b}}_{*},$$

which finishes the proof. □

The next result is given in ([42], Corollary 8.2).

**Lemma** **A3.**
*Suppose that $Z_{1},\ldots ,Z_{n}$ are i.i.d. random variables and there exists L>0 such that $C^{2}=E\exp\left(|Z_{1}|/L\right)$ is finite. Then for arbitrary u>0*

$$P\left(\frac{1}{n}\sum_{i=1}^{n}\left(Z_{i}-E\left[Z_{i}\right]\right)>2L\left(C\sqrt{\frac{2u}{n}}+\frac{u}{n}\right)\right)\leq \exp\left(-u\right).$$

**Lemma** **A4.**
*For arbitrary j=1,…,p and u>0 we have*

(A9) $$P\left(\frac{2}{n}\sum_{i=1}^{n}X_{ij}\left(X_{i}^{⊤}{\overset{˚}{b}}_{*}+E\left[Y\right]-Y_{i}\right)>16.4\sigma^{2}\left(3\sqrt{\frac{2u}{n}}+\frac{u}{n}\right)\right)\leq \exp\left(-u\right).$$

**Proof.** Fix j=1,…,p and u>0. Recall that $H{\overset{˚}{b}}_{*}=E\left[YX\right]$ and $E\left[X\right]=0.$ Thus, we work with an average of i.i.d. centred random variables, so we can use Lemma A3. We only have to find L,C>0 such that

(A10) $$E\exp\left(|X_{j}\left(X^{⊤}{\overset{˚}{b}}_{*}+E\left[Y\right]-Y\right)|/L\right)\leq C^{2},$$

where $X_{j}$ is the *j*-th coordinate of X. For each positive number a,b we have the inequality $ab\leq \frac{a^{2}}{2}+\frac{b^{2}}{2}.$ Therefore, we have

$$|X_{j}\left(X^{⊤}{\overset{˚}{b}}_{*}+E\left[Y\right]-Y\right)|\leq \frac{X_{j}^{2}}{2}+{\left(X^{⊤}{\overset{˚}{b}}_{*}\right)}^{2}+4.$$

Applying this fact and the Schwarz inequality we obtain

(A11) $$E\exp\left(|X_{j}\left(X^{⊤}{\overset{˚}{b}}_{*}-Y\right)|/L\right)\leq \exp\left(\frac{4}{L}\right)\sqrt{E\exp\left(\frac{X_{j}^{2}}{L}\right)E\exp\left(\frac{2{\left(X^{⊤}{\overset{˚}{b}}_{*}\right)}^{2}}{L}\right)}\;.$$

The variable $X_{j}$ is subgaussian, so using ([43], Lemma 7.4) we can bound the first expectation in (A11) by ${\left(1-\frac{2\sigma^{2}}{L}\right)}^{-1/2}$ provided that $L>2\sigma^{2}.$ The second expectation in (A11) can be bounded using subgaussianity of the vector $X_{T},$ ([43], Lemma 7.4) and Lemma A2 in the following way

$$E\exp\left(\frac{2{\left(X^{⊤}{\overset{˚}{b}}_{*}\right)}^{2}}{L}\right)\leq {\left(1-\frac{4\sigma^{2}}{L}\right)}^{-1/2},$$

provided that $4\sigma^{2}<L.$ Taking $L=4.1\sigma^{2}$ we can bound exp(4/L)≤2.7, because $H_{jj}=1$ implies that σ≥1. Thus, we obtain C≤3, where *C* is the upper bound in (A10). It finishes the proof. □

**Lemma** **A5.**
*Suppose that assumptions of Theorem 3 are satisfied. Then for arbitrary a∈(0,1),q≥1,ξ>1 with probability at least 1−Ka we have ${\bar{F}}_{q}\left(\xi \right)\geq F_{q}\left(\xi \right)/2,$ where K is an universal constant.*

**Proof.** Fix a∈(0,1),q≥1,ξ>1. We start with considering the $l_{\infty }$-norm of the matrix

(A12) $$\begin{array}{ccc} {\left|\frac{1}{n}{\tilde{X}}^{⊤}\tilde{X}-E\left[\tilde{X}{\tilde{X}}^{⊤}\right]\right|}_{\infty } & = & \max\left(\max_{j,k=1,\ldots ,p}\left|\frac{1}{n}\sum_{i=1}^{n}X_{ij}X_{ik}-E\left[X_{j}X_{k}\right]\right|,\right. \end{array}$$

(A13) $$\begin{array}{cc} \; & \left.\max_{j=1,\ldots ,p}\left|\frac{1}{n}\sum_{i=1}^{n}X_{ij}\right|\right). \end{array}$$

We focus only on the right-hand side of (A12), because (A13) can be done similarly. Thus, fix j,k∈{1,…,p}. Using subgaussianity of predictors, Lemma A3 and argumentation similar to the proof of Lemma A4 we have

$$P\left(\left|\frac{1}{n}\sum_{i=1}^{n}X_{ij}X_{ik}-E\left[X_{1j}X_{1k}\right]\right|>K_{2}\sigma^{2}\sqrt{\frac{\log(p^{2}/a)}{n}}\right)\leq \frac{2a}{p^{2}}\;,$$

where $K_{2}$ is an universal constant. The values of constants $K_{i}$ that appear in this proof can change from line to line. Therefore, using union bounds we obtain

$$P\left({\left|\frac{1}{n}{\tilde{X}}^{⊤}\tilde{X}-E\left[\tilde{X}{\tilde{X}}^{⊤}\right]\right|}_{\infty }>K_{2}\sigma^{2}\sqrt{\frac{\log(p^{2}/a)}{n}}\right)\leq K_{3}a.$$

Proceeding similarly to the proof of ([30], Lemma 4.1) we have the following probabilistic inequality

$${\bar{F}}_{q}\left(\xi \right)\geq F_{q}\left(\xi \right)-K_{2}\left(1+\xi \right)\left|T\right|\sigma^{2}\sqrt{\frac{\log(p^{2}/a)}{n}}\;.$$

To finish the proof we use (20) with $K_{1}$ being sufficiently large. □

**Proof of Theorem** **3.** Let a∈(0,1),q≥1,ξ>1 be arbitrary. The main part of the proof is to show that with high probability

(A14) $$|\hat{b}-b_{*}{|}_{q}\leq \frac{{\xi |T|}^{1/q}\lambda }{\left(\xi +1\right){\bar{F}}_{q}\left(\xi \right)}\;.$$

Then we apply Lemma A5 to obtain (22). Thus, we focus on showing that (A14) holds with high probability. Denote $A=\{|\nabla \bar{Q}\left(b_{*}\right){|}_{\infty }\leq \frac{\xi -1}{\xi +1}\lambda \}.$ We start with bounding from below probability of A. Recall that $b_{*}$ is the minimizer of $Q\left(b\right)=E{\left(1-Yb{\;}^{⊤}\tilde{X}\right)}^{2},$ which can be easily calculated, namely

$${\overset{˚}{b}}_{*}=H^{-1}E\left[YX\right]\;\mathrm{and}\;{\left(b_{*}\right)}_{0}=E\left[Y\right].$$

For every j=1,…,p the *j*-th partial derivative of $\bar{Q}\left(b\right)$ at $b_{*}$ is

(A15) $$\nabla_{j}\bar{Q}\left(b_{*}\right)=\frac{2}{n}\sum_{i=1}^{n}X_{ij}\left(X_{i}^{⊤}{\overset{˚}{b}}_{*}+E\left[Y\right]-Y_{i}\right).$$

The derivative with respect to the $b_{0}$ is

(A16) $$\nabla_{0}\bar{Q}\left(b_{*}\right)=\frac{2}{n}\sum_{i=1}^{n}\left(X_{i}^{⊤}{\overset{˚}{b}}_{*}+E\left[Y\right]-Y_{i}\right).$$

Taking λ, which satisfies (21), and using union bounds, we obtain that

(A17) $$P\left(A^{c}\right)\leq \sum_{j=0}^{p}P\left(|\nabla_{j}\bar{Q}\left(b_{*}\right)|>K_{2}\sigma^{2}\sqrt{\frac{\log(p/a)}{n}}\right).$$

Consider a summand on the right-hand side of (A17), which corresponds to j∈{1,…,p}. From (A15) we can handle it using Lemma A4. We just take u=log(p/a) and suffciently large $K_{2}.$ Probability of the first term on the right-hand side of (A17), which corresponds to j=0, can be bounded from above analogously as in the proof of Lemma A4. Proceeding is even easier, so we omit it. In further argumentation we consider only the event A. Besides, we denote $\theta =\hat{b}-b_{*},$ where $\hat{b}$ is a minimizer of a convex function (5), that is equivalent to

(A18) $$\left\{\begin{array}{ccc} \nabla_{j}\bar{Q}\left(\hat{b}\right)=-\lambda \mathrm{sign}\left({\hat{b}}_{j}\right) & \mathrm{for} & {\hat{b}}_{j}\neq 0;\\ |\nabla_{j}\bar{Q}\left(\hat{b}\right)|\leq \lambda & \mathrm{for} & {\hat{b}}_{j}=0;\\ \nabla_{0}\bar{Q}\left(\hat{b}\right)=0, \end{array}\right.$$

where j=1,…,p. First, we prove that θ∈C(ξ). Here our argumentation is standard [9]. From (A18) and the fact that ${\left|\theta \right|}_{1}=|\theta_{T}{|}_{1}+|\theta_{T^{c}}{|}_{1}+\left|\theta_{0}\right|$ we can calculate

$$\begin{array}{cc} 0 & \leq 2\theta^{⊤}{\tilde{X}}^{⊤}\tilde{X}\theta /n=\theta^{⊤}\left[\nabla \bar{Q}\left(\hat{b}\right)-\nabla \bar{Q}\left(b_{*}\right)\right]\\ \; & =\sum_{j\in T}\theta_{j}\nabla_{j}\bar{Q}\left(\hat{b}\right)+\sum_{j\in T^{c}}{\hat{b}}_{j}\nabla_{j}\bar{Q}\left(\hat{b}\right)-\theta^{⊤}\nabla \bar{Q}\left(b_{*}\right)\\ \; & \leq \lambda \sum_{j\in T}|\theta_{j}|-\lambda \sum_{j\in T^{c}}|{\hat{b}}_{j}{\left|+|\theta \right|}_{1}{\left|\nabla \bar{Q}\left(b_{*}\right)\right|}_{\infty }\\ \; & =\left[\lambda +|\nabla \bar{Q}\left(b_{*}\right){|}_{\infty }\right]|\theta_{T}{|}_{1}+\left[|\nabla \bar{Q}\left(b_{*}\right){|}_{\infty }-\lambda \right]|\theta_{T^{c}}{|}_{1}+|\theta_{0}||\nabla \bar{Q}\left(b_{*}\right){|}_{\infty }\;. \end{array}$$

Thus, using the fact that we consider the event A we get

$$|\theta_{T^{c}}{|}_{1}\leq \frac{\lambda +|\nabla \bar{Q}\left(b_{*}\right){|}_{\infty }}{\lambda -|\nabla \bar{Q}\left(b_{*}\right){|}_{\infty }}|\theta_{T}{|}_{1}+\frac{|\nabla \bar{Q}\left(b_{*}\right){|}_{\infty }}{\lambda -|\nabla \bar{Q}\left(b_{*}\right){|}_{\infty }}|\theta_{0}\left|\leq \xi \right|\theta_{\tilde{T}}{|}_{1}\;.$$

Therefore, from the definition of ${\bar{F}}_{q}\left(\xi \right)$ we have

$$|\hat{b}-b_{*}{|}_{q}\leq \frac{{\left|T\right|}^{1/q}{\left|{\tilde{X}}^{⊤}\tilde{X}\left(\hat{b}-b_{*}\right)/n\right|}_{\infty }}{{\bar{F}}_{q}\left(\xi \right)}\leq {\left|T\right|}^{1/q}\frac{|\nabla \bar{Q}\left(\hat{b}\right){|}_{\infty }/2+{\left|\nabla \bar{Q}\left(b_{*}\right)\right|}_{\infty }/2}{{\bar{F}}_{q}\left(\xi \right)}\;.$$

Using (A18) and the fact, that we are on A, we obtain (A14). □

### Appendix A.3. Results from Section 5

**Proof of Corollary** **2.** The proof is a simple consequence of the bound (22) with q=∞ obtained in Theorem 3. Indeed, for arbitrary predictors j∈T and k∉T we obtain

$$\begin{array}{ccc} |{\hat{b}}_{j}^{quad}| & \geq & |{\left(b_{*}^{quad}\right)}_{j}\left|-\right|{\hat{b}}_{j}^{quad}-{\left(b_{*}^{quad}\right)}_{j}|\geq b_{min}^{quad}-{\left|{\hat{b}}^{quad}-b_{*}^{quad}\right|}_{\infty }\\ & > & \frac{2\xi \lambda }{\left(\xi +1\right)F_{\infty }\left(\xi \right)}\geq |{\hat{b}}^{quad}-\left(b_{*}^{quad}\right){|}_{\infty }\geq |{\hat{b}}_{k}^{quad}-{\left(b_{*}^{quad}\right)}_{k}\left|=\right|{\hat{b}}_{k}^{quad}|. \end{array}$$

□

**Proof of Corollary** **3.** The proof is almost the same as the proof of Corollary 2, so it is omitted. □
